# Haemodynamic Responses to Selective Vagal Nerve Stimulation under Enalapril Medication in Rats

**DOI:** 10.1371/journal.pone.0147045

**Published:** 2016-01-14

**Authors:** Mortimer Gierthmuehlen, Thomas Stieglitz, Josef Zentner, Dennis T. T. Plachta

**Affiliations:** 1 Department of Neurosurgery, University Medical Center Freiburg, Freiburg, Germany; 2 Laboratory for Biomedical Microtechnology, Department of Microsystems Engineering-IMTEK, University of Freiburg, Freiburg, Germany; Fraunhofer Research Institution of Marine Biotechnology, GERMANY

## Abstract

Selective vagal nerve stimulation (sVNS) has been demonstrated to lower blood pressure (BP) in rats without causing major side effects. This method might be adapted for the treatment of therapy-resistant hypertension in patients. Converting enzyme inhibitors (CEIs) are among the first drugs that are administered for arterial hypertension and prominently reduce BP primarily by interacting with the renin-angiotensin system of the kidneys. Beyond the reduction of BP, CEI have a positive effect on the survival rate after myocardial infarction; they reduce the rates of stroke and improve the neurohormonal status in heart-failure patients. If sVNS might be introduced as a therapy against resistant hypertension, patients will at least partially stay on their CEI medication. It is therefore the aim of this study to investigate the influence of the CEI enalapril on the haemodynamic and respiratory effects of sVNS. In 10 male Wistar rats, a polyimide-based multichannel-cuff-electrode was placed around the vagal nerve bundle to selectively stimulate the aortic depressor nerve fibres. Stimulation parameters were adapted to the thresholds of the individual animals and included repetition frequencies between 30 and 50 Hz, amplitudes of 0.5 to 1.5 mA and pulse widths between 0.4 ms and 1.0 ms. BP responses were detected with a microtip transducer in the left carotid artery, and electrocardiography was recorded with subcutaneous electrodes. After intravenous administration of enalapril (2 mg/kg bodyweight), the animals’ mean arterial blood pressures (MAPs) decreased significantly, while the heart rates (HRs) were not significantly influenced. The effects of sVNS on BP and HR were attenuated by enalapril but were still present. We conclude that sVNS can lower the MAP during enalapril treatment without relevant side effects.

## Introduction

Arterial hypertension is a common disease that affects millions of patients. Despite medical therapy, up to 30% of these patients do not reach their target blood pressure (BP) of less than 140 mmHg [1]. In our effort to develop a neuromodulation treatment of therapy-refractory arterial hypertension, we recently proposed a technique of selective vagal nerve stimulation (sVNS) with a multichannel cuff electrode (MCE) that activates the baroreflex in rats [2]. Without surgically dissecting the vagal baroreceptive fibres from the surrounding tissue (i.e., the aortic depressor nerve, ADN), the MCE is wrapped around the vagal nerve bundle including the vagus, the ADN, the laryngeal recurrent nerve, the small supply vessels and some connective and fat tissue. A specialized algorithm locates the baroreceptive fibres inside the vagal nerve bundle and focuses the stimulation to selectively activate the baroreflex. We demonstrated reductions in arterial blood pressure with minimal side effects during sVNS in male Wistar rats with no medical treatment.

Converting enzyme inhibitors (CEIs) are among the primary drugs that are used to treat arterial hypertension and reduce BP by interacting with the renin-angiotensin system of the kidneys. CEIs have also been demonstrated to reset the baroreflex in rats toward a lower arterial blood pressure [3]. In addition to the BP reduction capabilities, CEI also feature further beneficial effects on hypertensive patients. They positively influence the remodelling of the cardiac muscles after myocardial infarction and modify the hormonal system. Additionally, they reduce the occurrence of stroke and mortality in heart failure patients [4]. Due to these positives properties of CEI it can be assumed that sVNS will might be used as an additional antihypertensive therapy rather than a monotherapy. In the context of the above-mentioned observation that CEI medications possibly influence the baroreflex, the aim of the present study was to elucidate whether intravenously administered enalapril interferes with antihypertensive sVNS in a male Wistar rat model.

## Materials and Methods

This study was approved by the Regierungspraesidium Freiburg, Baden-Wuerttemberg and the Ethics Committee of the University of Freiburg (G13-44), and we adhered to the Principles of Laboratory Animal Care of the National Institutes of Health. The system setup and signal analysis have previously been described in detail [2]. Here, we used the following electrodes and technical and surgical methods to conduct the study.

### Multichannel cuff electrode (MCE) and pressure transducer

Established micromachining technologies were applied to manufacture flexible multichannel cuff-electrodes [5]. We used a multichannel cuff electrode (MCE) with 24 electrodes arranged in eight tripoles around the cuff perimeter with 45° spacing. The basic configuration has previously been described [6]. The three electrode rings recorded the signals of the vagal nerve bundle, and two electrodes facing outward served as reference contacts. The length of the MCE was 10 mm, its inner diameter was designed to be 0.8 mm, and the longitudinal distance between the electrodes was 2 mm. The micromachined contacts and interconnection lines were sandwiched between two layers of polyimide with a total thickness of 11 μm. The 300-nm sputtered platinum thin film metallizations were coated with 1000 nm of sputtered iridium oxide (SIROF) on the electrode sites. The electrocardiogram (ECG) was recorded with three needle electrodes placed subcutaneously on the right and left chest and the left foot. A 1.5 French (F) microtip pressure transducer (Millar Instruments, Houston, Texas, USA) was placed in the left carotid artery for invasive blood pressure (BP) monitoring. The microtip catheter was connected to an in-house constructed amplifier. The amplified BP signal was fed to the analogue input of the data acquisition system. The ECG and BP data were digitized with identical parameters on separate input channels of the setup.

### Surgical Procedure

Ten male Wistar rats (bodyweights (BWs) 370–525 g, mean 452.5 g) were anaesthetised with 2–4 vol.% isoflurane (Abbott, USA). Analgesia was achieved with carprofen (Rimadyl^®^, Pfizer, USA, 5 mg/kg BW s.c.). Anaesthesia was maintained with 1–2 vol.% isoflurane that was regulated according to the respiration rate. During the experiments, the rats were placed on a temperature-controlled, electrically isolated heat pad (Harvard Apparatus, USA) and received an intravenous catheter in the tail vein. Fluid replacement was achieved with a saline solution at a rate of 1 ml/100 g BW per hour. Under microscopic examination (VM900, Moeller-Wedel GmbH, Germany), we approached the left neurovascular sheath through a ventral neck incision and ligated the left carotid artery distally. An aneurysm-clip temporarily blocked the carotid artery proximally. Through a small incision, a 1.5 F microtip transducer was inserted into the carotid artery and advanced until the Waynforth position was reached [7]. Two ligations secured the catheter. The MCE was connected to the data acquisition system and gently placed around the vagal nerve bundle.

### Data acquisition and localization of the baroreceptive fibres

Electroneurograms and the ECG were recorded with a PZ3 system (Tucker Davis Technology, Florida, USA) and an RZ2-module connected to a PC. The PZ3 operates with a dynamic amplification (up to 60 dB) that automatically adapts the gain of the given signal strength and matches it to the voltage range (+/- 3 mV chosen). All monopolar signals from each of the 24 electrodes and the 2 reference electrodes were amplified under the prevailing conditions and sampled at a rate of 12 kHz. All input signals were notch-filtered (50 Hz), and the recorded and digitized signals were band-pass filtered using MATLAB (Butterworth 2nd order filter, 20 to 200 Hz). The surgeries and experiments were performed in a faraday cage made of copper wires. The cage and all experimental devices were connected to a common ground. As previously described [8], the tripole contacts located near the baroreceptive fibres (ADN) were identified through coherent averaging, and these contacts were chosen for the stimulation procedure described below.

### Selective vagal nerve stimulation and drug administration

We performed current-controlled stimulation with charge-balanced biphasic rectangular pulses that were generated by the RZ2 module and fed into an in-house constructed 8-channel voltage-to-current converter. The D/A conversion rate was 24 kHz, and the resolution was 16-bit. The centre electrode of the chosen tripole was the cathode, and the two large peripheral ring electrodes functioned as the anodes. After localization of the blood pressure-related tripole, we proceeded to apply each combination of stimulation parameters three times in an arbitrary order. All stimulations contained trains of 100 pulses and were performed with the following parameters: repetition rates of 30 Hz, 40 Hz and 50 Hz; stimulation amplitudes of 0.5 mA, 0.6 mA, 0.8 mA, 0.9 mA, 1.0 mA, 1.3 mA and 1.5 mA; and pulse widths of 0.4 ms, 0.6 ms, 0.8 ms and 1.0 ms. The individual stimulation trains were separated by an interval of at least 13 seconds. Stimulation amplitudes exceeding 1 mA were applied only in 5 of the 10 test animals.

We observed large inter-individual variance in the responses to certain stimulation parameter settings. Therefore, we intentionally stopped increasing the stimulation amplitude if the BP reduction observed at a given setting exceeded 50% of the start value.

**Fig 1:**
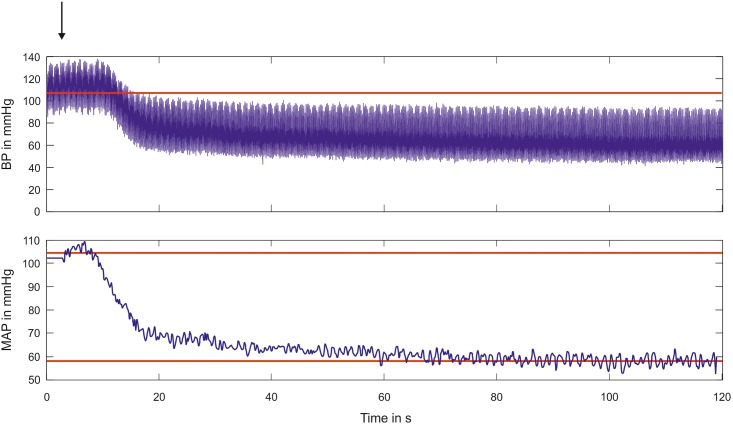
Acute effects of the administration of enalapril on BP and MAP. The upper plot illustrates the BP reduction induced by the administration of enalapril. After a rapid decline in the first 10 seconds after application, the development of an equilibrium state required more than 100 seconds. Because the PA oscillation obscured the observation of the BP, the lower plot illustrates the calculated MAP values. The remaining oscillation visible in the lower plot was due to respiration. The red line in the upper plot and the upper red line in the lower plot mark the average MAP of 106 mmHg prior to administration of enalapril. The red line in the lower plot indicates the steady state of the MAP (at 58 mmHg). The black vertical arrow indicates the moment of enalapril application.

Following the initial recording (localization) and stimulation, 2 mg/kg BW enalapril (Enahexal^®^, Hexal, Germany) was intravenously administered over a period of 30 seconds Fig 1). The rats were allowed to adapt to this medication for 5 min. Next, the recording and stimulation procedures were applied again with the same parameters.

### Experimental procedure, data analysis and statistics

**Fig 2:**
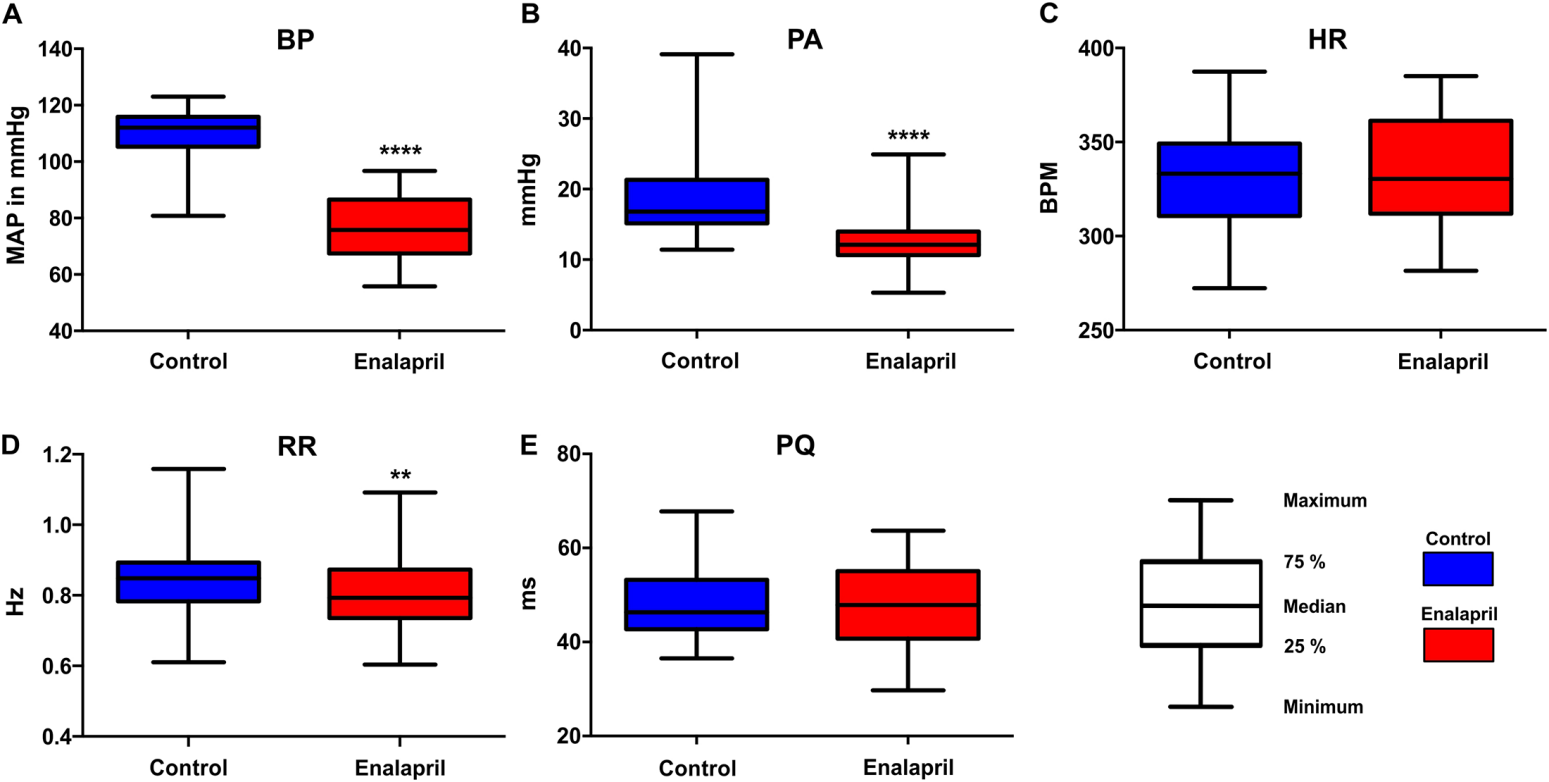
Effects of enalapril on BP (MAP) in A), PA in B), HR in C), RR in D) and PQ-time in E). All box plots present the median as the horizontal line, the centre quartiles as the box-frames and the maximums and minimums as the whiskers. While the BP, PA and RR exhibited significant reductions following the application of Enalapril, the HR and the PQ-time remained unaffected.

**Fig 3:**
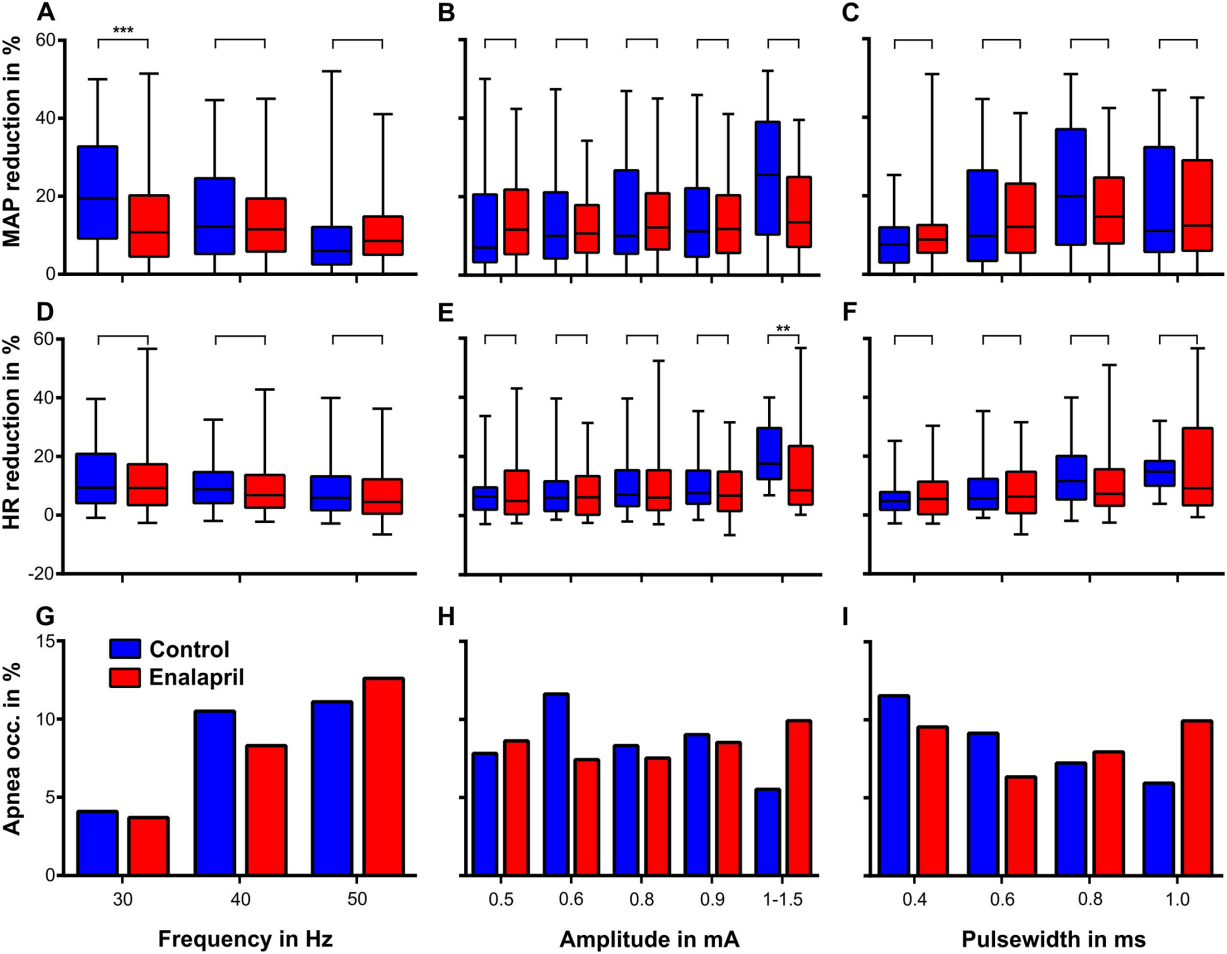
These plots show the influence of selective VNS on blood pressure (A to C), heart rate (D to F) and the occurrence of apnoea (G to I) as functions of the stimulation parameters (i.e., frequency, amplitude and pulse width) before (blue boxes, N = 10) and after administration of enalapril (red boxes, N = 10, 2 mg/kg). The values were obtained during the stimulation with 100 pulses, which means during 3,3 s at 30 Hz, 2,5 s at 40 Hz and 2 s at 50 Hz, with additional five seconds of recording immediately after the last stimulation pulse. We observed significant effects of enalapril on the post-stimulation results in only two cases, i.e., at the 30-Hz stimulation rate, the BP reduction was significantly smaller, and at amplitudes between 1 and 1.5 mA, the HR reduction was also significantly smaller. For further explanation, see the text.

Each experiment began with a 10-minute recording of all of the channels of the MCE. This baseline recording was followed with an automatized stimulation series involving alternating stimulation parameters. After this period, enalapril was administered, and the BP, ECG and MCE signals were recorded for another 10 minutes. Once the MAPs did not exhibit any further decreases, we began the second round of the stimulation sequence; this round was conducted under the enalapril regime and with the identical values and order of the parameter settings. The prolonged baseline recordings enabled the localization of the baroreceptive fibres and enabled the collection of the average blood pressure (BP), blood pressure amplitude (PA), heart rate (HR), respiration rate (RR) and PQ time of the electrocardiogram values. The BP, PA, HR and RR values were extracted from the BP recordings from the microtip catheter, and the PQ-intervals were collected from the ECG recording via an in-house-written MATLAB script. Two weaker ECG signals were enhanced using a Wiener filter. The PQ-interval analysis was not accessible during the stimulation periods because the stimulation artefact was superimposed on the ECG. The stimulation-related blood pressure reductions were normalized to the original MAP and are reported as percentages. This normalization was necessary to maintain the cross-compatibility between the BP decreases before and after the enalapril administrations because enalapril reduces the pressure amplitude (PA). The HR data were processed in the same manner. The stimulation driven apnoea was not measured as a rate because if post-stimulus apnoea occurred at all, it was only for a single respiration cycle. Because all stimulation combinations were tested five times, we counted the total number of apnoea occurrences for every set of stimulation parameters. The post-stimulation BP and HR reductions were processed as multivariate parameters and pooled for the ten test animals. For the statistical analyses, the multivariate variables of the stimulus-driven decreases in BP and HR were binned according to the stimulation parameters of frequency, amplitude and pulse width. The distributions of these bins were then tested for normality using the D’Agostino omnibus normality test and the Shapiro-Wilk test. These tests of the normalization of the binned data were performed independently before and after the enalapril stimulus-driven BP and HR decreases. Because the majority of the bins failed to exhibit normal distributions, non-parametric Kruskal-Wallis test and Dunn’s test for multiple comparisons were selected to compare the different bins. The PQ intervals of each animal were drawn from four different time intervals (each at least 5 minutes) that did not contain any stimulation. The intervals were as follows: A) before stimulation and drug administration, B) immediately after selective stimulation, C) during the application of enalapril, and D) after stimulation in the enalapril condition. The PQ intervals of these intervals were pooled and compared using the Kruskal-Wallis test and Dunn’s test for multiple comparisons. All box plots (Figs 2 and 3) present the median values with the two inner quartiles indicated by the box frame. The whiskers present the minimum and maximum values. The significance levels are indicated according to the conventions: *, p = 0.05–0.01; **, p = 0.01–0.001; ***; p = 0.001–0.0001; and ****, p < 0.0001.

## Results

### Baselines heart rate, respiration rate, pulse amplitude and mean arterial pressure readings before and after the administration of enalapril

The average time until the BP reached a steady level after the application of enalapril was 120.1 s (STD 28.8 s, Fig 1). For the comparison of the control against enalapril, the vital parameters of all of the animals (n = 10) were recorded for 10 minutes at the beginning of the experiment (before stimulation and drug application) and after a steady state was established (before stimulation). The results from all 10 animals were pooled and tested for normality using the Shapiro-Wilk test. Because normality could not proven for all of the datasets, the control and enalapril data were compared in terms of each vital parameter using two-tailed, non-parametric Mann-Whitney tests (Fig 2). The MAP was significantly reduced (p<0.0001) by enalapril (Figs 1 and 2A) from a mean of 110.0 mmHg (median 112 mmHg, STD 8.23 mmHg) to 75.28 mmHg (median 75.8 mmHg, STD 11.35 mmHg). The PA (Fig 2B) significantly decreased (p<0.0001) from a mean of 18.7 mmHg (median 16.8 mmHg, STD 5.44 mmHg) to 12.64 mmHg (median 12.1 mmHg, STD 3.24 mmHg). The HR (Fig 2C) was not significantly altered by enalapril (p = 0.086) and exhibited a slight increase from 329.7 beats per minute (BPM; median 333.1 BPM, STD 27.83 BPM) to 333.9 BPM (median 330.4 BPM, STD 27.75 BPM). The RR (Fig 2D) was reduced from a mean of 0.85 Hz (median 0.85 Hz, STD 0.11 Hz) to 0.81 Hz (median 0.79 Hz, STD 0.10 Hz). This RR reduction (Fig 2E) was significant (p = 0.0014). The PQ time changed from 47.75 ms (median 46.32 ms, STD 0.0078 ms) to 47.40 ms (median 47.88, STD 0.008 ms) and this alteration was non-significant (p = 0.3).

### BP response to electrical stimulation before and after the administration of Enalapril

All stimulation trials prior to enalapril administration were pooled independently of any other stimulation parameter across the stimulation frequencies (30 Hz, 40 Hz and 50 Hz). In the first step, the control results were compared with the enalapril results. In the second step, the frequency-dependent changes within each condition were statistically analysed. The control vs. Enalapril comparison yielded a significantly (p<0.001) blunted effect of the baroreflex with stimulations of 30 Hz. All other stimulation frequencies (40 Hz and 50 Hz) failed to elicit significantly different sVNS responses (Fig 3A). The comparison within the control results revealed a significant decrease in the effect on BP reduction with increasing frequency (30 vs. 40 Hz p<0.05, 40 vs. 50 Hz p<0.01, 30 vs. 50 Hz p<0.001). After enalapril administration, no trends and no significant differences were found across the three stimulation frequencies (Fig 3A). Enalapril did not significantly alter the correlation between the stimulation amplitude and the strength of the baroreflex (Fig 3B). There appeared to be a trend toward an increased baroreflex with increasing stimulation amplitude. However, this trend never reached significance with one exception: within the control results, the MAP reductions observed at amplitudes between 1–1.5 mA were significantly greater than those of the control group at 0.9 mA (p<0.05), but these results were not significant when compared to the enalapril results at 0.9 mA and 1–1.5 mA (Fig 3B). On average, increasing the pulse width elicited an increased baroreflex in both the control and enalapril conditions (Fig 3C). This increase stopped at the pulse width of 0.8 ms. Pulse widths of 1 ms triggered smaller decreases in blood pressure. At no point did we observe a significant difference between the responses before and after the administration of enalapril. However, these increments of pulse width and the correlated increments in blood pressure reduction were significant within the control group (0.4 ms vs. 0.6 ms, p<0.05; 0.6 ms vs. 0.8 ms, p<0.05; Fig 3C).

**Fig 4:**
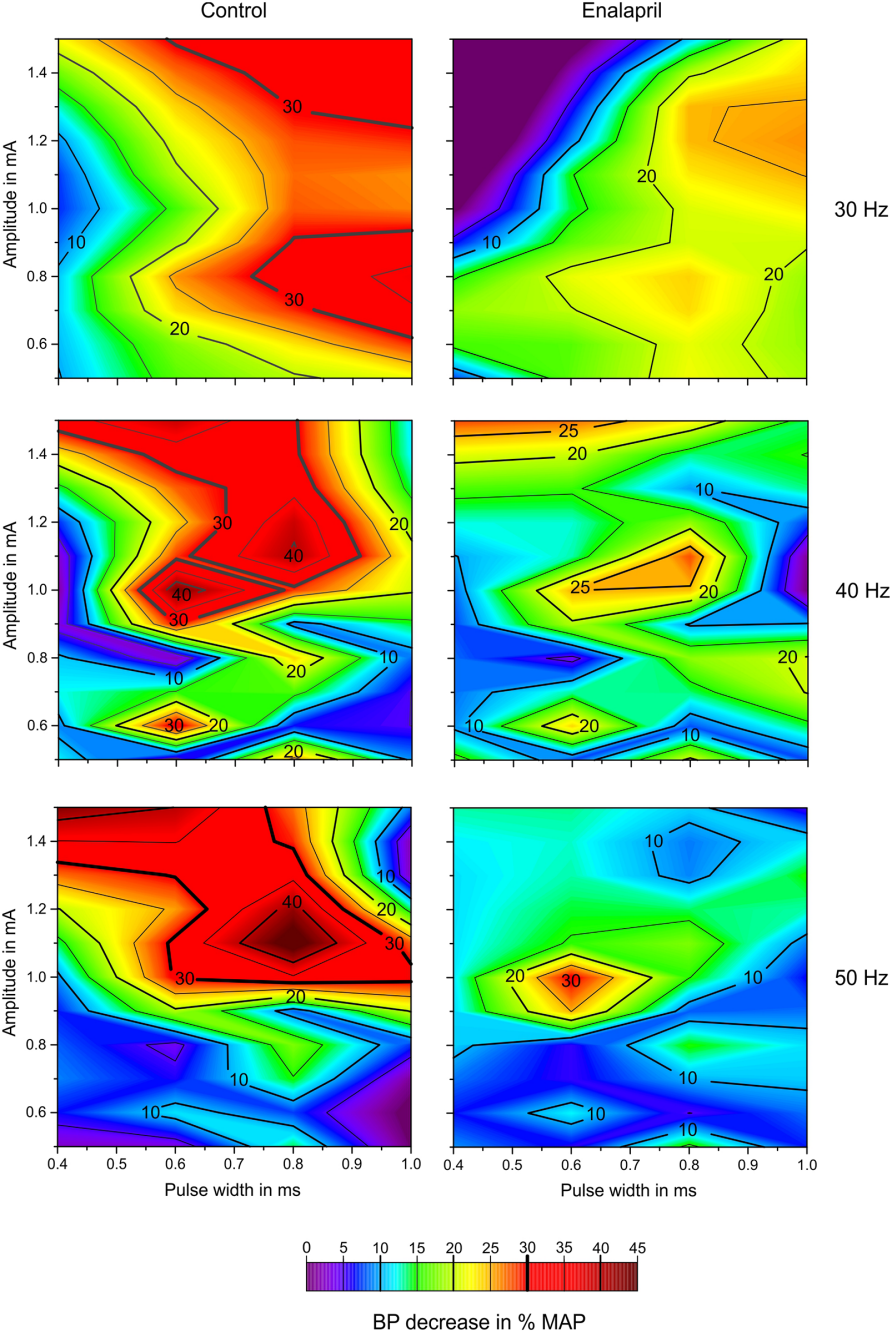
Contour plot of the BP reductions (%). The abscissa represents the pulse widths; the ordinate provides the amplitude. In the first row, the stimulation frequency is 30 Hz, in the middle row 40 Hz and in the lower row 50 Hz. These contour plots are “heat maps” to visualize the averaged (over all animals) influence of multiple parameters on the BP. Red zones stand for a high percentile reduction of BP, while blue and purple zones represent less or no change in BP. The left column shows the results of sVNS before administration of enalapril, the right column shows the results of sVNS after administration of enalapril. It becomes noticeable that, especially in the center row, the heat maps have a similar shape, but the effectiveness of BP reduction is less after enalapril is given. Apparently, enalapril seems to attenuate the baroreflex without changing the actual underlying mechanisms.

Although the contour plots display smoothened and interpolated results, they still offer a good opportunity to present an overview of the effects of the given stimulation combinations and how they compared with other settings (Fig 4). Comparison of the two columns of Fig 4 (left column control, right column enalapril) at the given stimulation frequencies, obviously reveals that increasing the stimulation parameters toward larger charges did not automatically increase the strength of the triggered baroreflex. Unlike typical strength-duration correlations, increasing the amount of charge/cycle/phase applied to the nerve resulted in BP reduction “hot spots” of very potent stimulation combinations. The correlation between stimulus frequency and the minimum current necessary to elicit a BP effect can also be observed in both scenarios (i.e., control and enalapril). Higher frequencies required larger threshold currents to achieve an average BP reduction of 10% and more.

### HR response to electrical stimulation before and after the administration of enalapril

Without enalapril application, increasing the stimulation frequency resulted in a less pronounced bradycardic response after stimulation. This trend was not influenced by enalapril. No significant deviations were found between the control and the enalapril results (Fig 3D). Within the groups (i.e., control and enalapril), in both cases, significant differences were found between the 30-Hz and 50-Hz stimulation frequencies (both p<0.01). The stimulation amplitude slightly but non-significantly correlated with the HR reduction. This pattern held for both the control and enalapril groups. This effect was emphasized at the amplitudes of 1–1.5 mA at which a significant difference between the HR reductions in the control and enalapril results was found (p<0.01). In both groups, the bradycardia elicited at 1–1.5 mA was significantly greater than that observed in any of the other within-group results (Fig 3E). On average, increasing the pulse width also resulted in a slight increase in bradycardia. For higher PWs above 0.8 ms, this effect became stronger (Fig 3F). We observed this trend in both scenarios, which resulted in a lack of a significant difference between the control and enalapril results. These “step” increases in bradycardia at pulse widths of 0.8 ms and longer were significant within both groups (p<0.01), whereas the results below 0.8 ms did not exhibit any significant differences. The same pattern held for the two sets of results at and above 0.8 ms.

**Fig 5:**
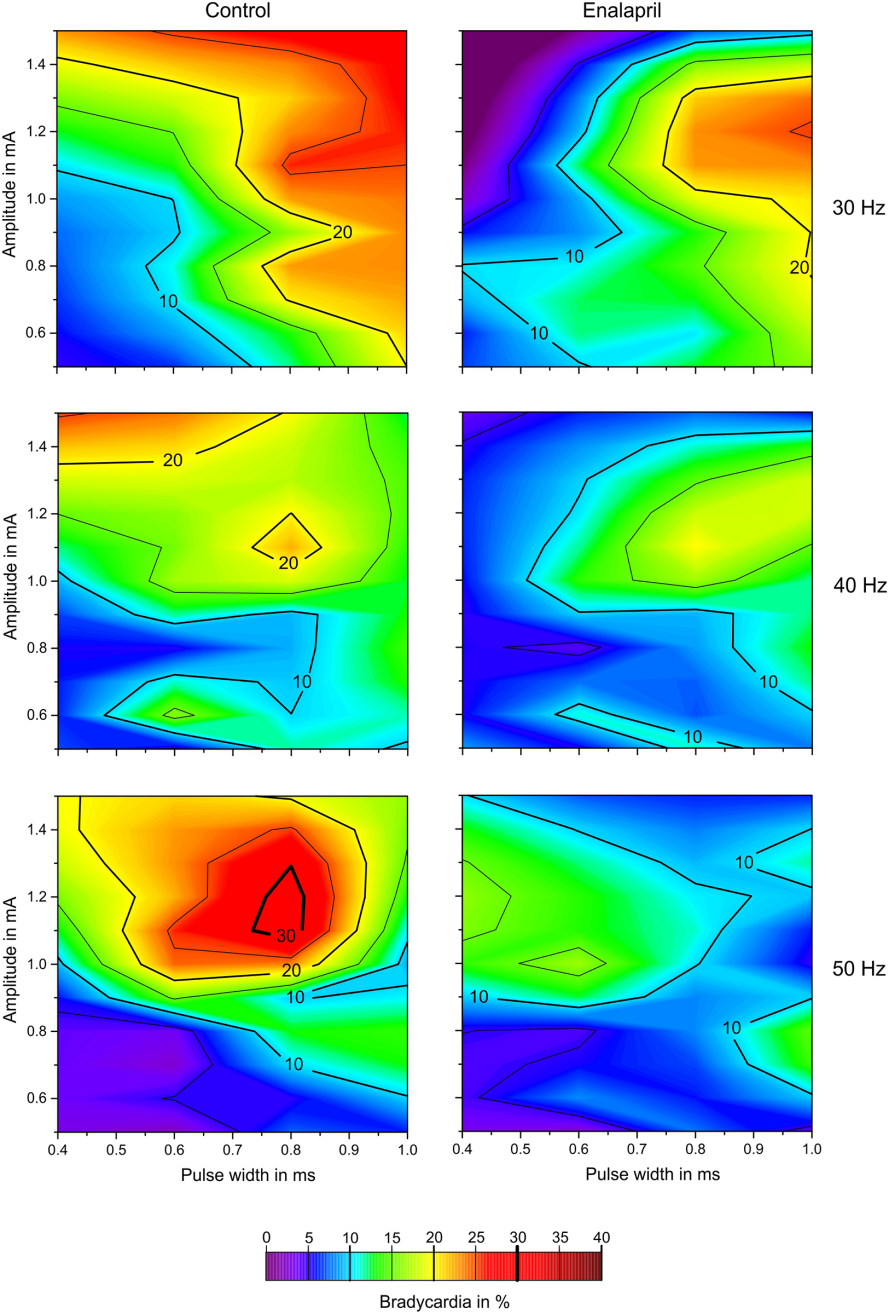
Contour plot of the HR reductions (%). Similar to Fig 4, the x-axis shows the pulse widths and the y-axis the amplitude. The stimulation frequency is 30 Hz in the first row, 40 Hz in the center row and 50 Hz in the lower row. This heat map visualizes the influence of the stimulation parameters on the change of HR. Red zones stand for a high percental reduction of HR, while blue and purple zones represent less or no change in HR. The left column shows the results of sVNS before administration of enalapril, the right column shows the results of sVNS after administration of enalapril. Note that—even more then within the contour plots of BP in Fig 4—the shape of the heat maps does not change after administration of enalapril. The magnitude of HR changes is very different before and after this drug is applied (left vs. right column). This again puts emphasizes on the theory that enalapril seems to attenuate the baroreflex without changing its actual underlying mechanisms.

The contour plot of the stimulus-related bradycardia (Fig 5) demonstrates the benefits of having all information available compared with integrating over one variable. Regarding the single variables (Fig 3), a non-significant trend was observed across the stimulation frequencies; i.e., lower frequencies were associated with greater overall bradycardia. The distributions of the 30-Hz results (within conditions) were significantly different from those at 50 Hz. Comparison of this finding with the contour plots provides support for the notion that if the stimulation actually triggers the baroreflex, the levels of bradycardia reach quite high values at the 50-Hz stimulation frequency.

### RR responses to electrical stimulation before and after the administration of enalapril

The numbers of occurrences of apnoea were counted after each of the stimulations. On average, the primary BP depressing effect following each stimulation lasted approximately 5 seconds. With typical RRs below 1 Hz, we did not expect to observe more than two occurrences of apnoea per stimulation trial, which is too low to obtain a rate. Indeed, we never observed more than one occurrence of apnoea following a stimulation. The overall incidence of apnoea was lower in the enalapril condition than the control condition. Increased stimulation frequency (Fig 3G) resulted in an increase in the occurrence of apnoea, and the absolute maximum (15%) was observed in the enalapril group at 50 Hz.

The stimulus amplitude had an inhomogeneous effect on the occurrence of apnoea. The absolute maximum was observed at 0.6 mA in the control group. Weaker and stronger stimulations had a far less influence on the occurrence of apnoea. To some extent, the results under enalapril were an inversion of the results in the control condition. Under enalapril, the lowest apnoea count was observed at 0.6 mA, and higher counts were observed with lower and higher stimulus amplitudes (Fig 3H). The pulse width had an almost linear influence on the occurrence of apnoea in the control condition; increasing pulse widths elicited steady 5% step decreases in the occurrence of apnoea. In the enalapril-treated animals, the relationship between pulse width and apnoea was rather heterogeneous. The absolute minimum was observed at 0.6 ms of PW. Shorter and longer PWs resulted in clear increases in the occurrence of apnoea (Fig 3I).

### PQ time responses to electrical stimulation before and after the administration of enalapril

A Dunn’s multiple comparison test did not detect any significant difference in the PQ times before and after stimulation in the control or enalapril conditions. However, stimulation lead to an obvious but slight increase in the PQ time (Table 1).

**Table 1:** PQ times with and without enalapril application pre- and post-stimulus.

	A) Pre Stimulus Control (n = 10)	B) Post Stimulus Control (n = 4)	C) Pre Stimulus Enalapril (n = 8)	D) Post Stimulus Enalapril (n = 7)
Mean	48 ms	47 ms	48 ms	47 ms
STD	7 ms	9 ms	5.5 ms	10 ms
Normal Dist.	no	no	yes	no

## Discussion

Antihypertensive neuromodulation techniques were proposed as early as the 1960s, when Bilgutay and Neistadt suggested that electrical stimulation of the sinus caroticus could activate the baroreflex and reduce BP [9–11]. Although the technique itself was successful in decreasing the BPs of several patients over a period of years, new medical therapies and technical limitations decelerated the development of such sinus caroticus stimulation (SCS) procedures until the 2000s. At that time, a modern stimulation device (Rheos^®^) was developed and introduced into clinical trials over the following years. Although SCS was able to permanently reduce BP by 30 mmHg and therefore demonstrated the general suitability of the baroreflex for chronic antihypertensive stimulation, the periprocedural complication rate was as high as 25% [12, 13]. Therefore, we investigated whether focused vagal nerve stimulation—a technique that has been widely applied in the treatment of epilepsy for over 15 years with a low complication rate [14]—could activate the baroreflex without causing vagus-associated side effects such as severe bradycardia. We described the technique of selective vagal nerve stimulation (sVNS) in a male Wistar rat model in which we were able to reduce BP without major side effects [2].

Such sVNS might therefore be a therapeutic option for patients with resistant hypertension—a severe condition in which patients do not reach their target BP despite medical treatment with three or more antihypertensive drugs. One must keep in mind that many of these drugs exhibit other positive side effects on the cardiovascular and renal system—CEI for instance reduce the mortality and stroke rate in heart failure patients by influencing the hormonal system and interfering with the cardiac remodelling process after infarction [4]. It is therefore very likely that even if sVNS becomes available as a therapeutic option against resistant hypertension, patients will probably stay at least on some of their antihypertensive drugs. We therefore strived to investigate the interaction of CEI with sVNS.

The antihypertensive effects of converting enzyme inhibitors (CEIs) are thought to be mediated by both a peripheral effect and direct central effect in the baroreflex centre of the brainstem because the intravenous administration of CEIs decreases the mean arterial pressure (MAP) without causing reflex tachycardia [15]. In our study, the HRs exhibited an average increase of 1% following the administration of a CEI. Although this change was not significant, a trend in the direction of tachycardia was present, which agrees with other studies have found increases in HR following the administration of enalapril [16–18]. However, in general, the influence on HR seems to be irrelevant to the antihypertensive effects of CEIs [3, 19]. Although there are slight differences between the two commonly used CEIs, i.e., enalapril and captopril, regarding renal and mesenteric blood flow [20], their haemodynamic effects seem to be comparable [21–23].

CEIs have been demonstrated to reset the baroreflex in rats toward lower arterial blood pressures [3, 15]. The activation of the baroreflex by electrical stimulation of the aortic depressor nerve (ADN) is blunted in urethane-anaesthetised Wistar rats following the intravenous administration of captopril [21]. The activation of baroreceptive axons triggers a comprehensive, multistage neuronal network with secondary control mechanisms that are primarily centred in the nucleus solitarii (NTS) of the brain stem. The effects we measured (i.e., BP, PA, HR, RR, and PQ time) are therefore not direct functions of the activation energy but rather involve a secondary mechanism and that alters the responsiveness of the entire baroreflex system that we were unable to monitor.

As mentioned above, Takeda’s observation agrees with our findings; these authors utilised stimulation frequencies only up to 30 Hz, and we observed a significantly blunted haemodynamic effect when sVNS was performed at 30 Hz. All frequencies above 30 Hz elicited a nearly unaltered tendency in the baroreflex before and after enalapril application. Notably, enalapril blunted the frequency-dependent activation of the baroreflex.

### Effects of sVNS and enalapril on BP and HR

The peak BP reduction was observed at a pulse width of 0.8 ms prior to the application of enalapril. Following enalapril application, larger pulse elicited larger decrease in BP, but this effect seemed to be attenuated. The contour plots of 40 Hz and 50 Hz feature dedicated “hot spots” that seemed to drive the baroreflex in an ideal manner (Fig 4). Comparison of the heat map patterns (for the individual frequencies) of the control and the enalapril conditions indicated that the plots featured similar contour lines. The overall intensities of the control and enalapril conditions were different, which might reflect the reduced potential remaining for the baroreflex to unfold in the enalapril conditions. This observations might support the assumption that enalapril attenuates the baroreflex but does not alter its inherent mechanisms.

Stimulation amplitudes of 1–1.5 mA elicited significantly different effects on HR before and after the application of enalapril (Fig 5). Prior to the administration of the drug, stimulation at 1–1.5 mA caused a highly significant increase in bradycardia, whereas after enalapril administration, the bradycardic response was also significant but was mild relative to the increase observed in the control condition. The reason for these findings remains unclear, but it is interesting to note that enalapril seemed to exhibit both a “pro-tachycardic” and a blunting effect regarding the baroreflex. The distributions of “hot spots” (Fig 5) were again different for the different stimulation frequencies. The “hot spots” of bradycardia correlated with the hot spots displayed in Fig 4; stronger baroreceptive effects were associated with stronger bradycardic side effects. Again, enalapril clearly dampened the effect of bradycardia but did not alter the peaks, which suggests that the underlying mechanisms were not changed per se.

Comparing Figs 4 and 5, the stimulation frequency of 40 Hz seems to be most efficient at a pulse width of 0.6 ms and a stimulation amplitude of 1.0 mA in reducing the BP by over 40%. The same set of parameters does not provoke a bradycardia of more then 15%, suggesting that this parameter combination—with a special focus on the stimulation frequency—might be the most convenient in reducing BP.

### Effects of sVNS and enalapril on RR, apnoea occurrence and PQ time

The RR was significantly decreased following the administration of enalapril; this effect was probably mediated by the parasympathomimetic properties of the CEI. Before the application of enalapril, the occurrence of apnoea during sVNS was significantly influenced by the stimulation frequency but not by the pulse amplitude. These findings agree with those from the study McMullan et al. [24]. These authors investigated the effect of ADN stimulation on RR and also observed a slight decrease in RR during stimulation. It must be mentioned that McMullan investigated vagotomized rats only with a stimulation frequency of 50 Hz, a 3-ms pulse width and a constant voltage, whereas we used many different parameters and constant current stimulation. Prior to enalapril, the pulse width was negatively correlated with the respiration rate. The frequency-dependent influence was slightly blunted by enalapril. The peak occurrence of apnoea was observed at an amplitude of 0.6 mA prior to enalapril administration, but this peak was absent following enalapril administration, and the amplitude no longer seemed to have any influence on apnoea. It was very noticeable that the effect of the pulse width on the occurrence of apnoea was almost inverted; prior to enalapril administration, the highest occurrence of apnoea was observed at small widths, whereas following enalapril administration, the numbers of apnoea occurrences increased with pulse width. The reason for this observation remains unclear but could possibly be related to a reduction in parasympathetic tone and a consequent reduction in parasympathetic reserve that would promote apnoea. The absence of a significant prolongation of the PQ time indicates that the selective vagal nerve stimulation did not significantly activate the cardiac efferent fibres.

## Conclusion

Enalapril blunts the effects of selective vagal nerve stimulation on blood pressure and heart rate but does not seem to affect the actual underlying mechanisms. Selective VNS remains a therapeutic option even during enalapril regimens. Enalapril reduces the frequency-dependent reduction in BP during sVNS and the BP-lowering effect of the pulse width. In contrast, the occurrence of apnoea during selective vagal nerve stimulation was significantly influenced; without enalapril, the occurrence of apnoea increased with increasing pulse width, and this effect was almost inverted by enalapril. The reasons for these findings remain unclear. Although enalapril reduced the specific influence of the stimulation frequency and the pulse width on the reduction of BP, sVNS still lowered the BP under enalapril without causing different side effects. As illustrated by the heat maps in Figs 4 and 5, the effects of sVNS on BP and HR stayed comparable before and after application of enalapril, which mostly seemed to blunt—and not change—the mechanisms underlying the baroreflex. The present study suggests that enalapril does not cause relevant interference with the proposed antihypertensive effects of vagal nerve stimulation.

The technique of selective vagal nerve stimulation has been demonstrated to be feasible in a rat model and will now be transferred to a large animal model, i.e., sheep. Sheep have a larger vagal anatomy in which the ADN is not separate from but is rather merged with the vagal nerve. The anatomy of the sheep model is much closer to the human anatomy. Steering currents and anodal blocks represent additional options for high-resolution electrodes on the perimeters of cuff electrodes to achieve highly selective stimulation. These methods will be evaluated to enable even more focused and selective nerve stimulation to reduce side effects to the fullest possible extent.
